# Activation of matrix metalloproteinases following anti-Aβ immunotherapy; implications for microhemorrhage occurrence

**DOI:** 10.1186/1742-2094-8-115

**Published:** 2011-09-09

**Authors:** Donna M Wilcock, Dave Morgan, Marcia N Gordon, Tiffany L Taylor, Lisa A Ridnour, David A Wink, Carol A Colton

**Affiliations:** 1 University of Kentucky Sanders-Brown Center on Aging, Department of Physiology, Lexington KY 40536 USA; 2 Duke University Medical Center, Department of Medicine, Division of Neurology, Durham NC 27710 USA; 3 University of South Florida, Department of Molecular Pharmacology and Physiology, Tampa, FL 33612 USA; 4 National Cancer Institute, Radiation Biology Branch, Bethesda, MD 20892 USA

**Keywords:** Immunotherapy, matrix metalloproteinases, inflammation, microhemorrhage, amyloid, cerebral amyloid angiopathy, transgenic mouse, Alzheimer's disease

## Abstract

**Background:**

Anti-Aβ immunotherapy is a promising approach to the prevention and treatment of Alzheimer's disease (AD) currently in clinical trials. There is extensive evidence, both in mice and humans that a significant adverse event is the occurrence of microhemorrhages. Also, vasogenic edema was reported in phase 2 of a passive immunization clinical trial. In order to overcome these vascular adverse effects it is critical that we understand the mechanism(s) by which they occur.

**Methods:**

We have examined the matrix metalloproteinase (MMP) protein degradation system in two previously published anti-Aβ immunotherapy studies. The first was a passive immunization study in which we examined 22 month old APPSw mice that had received anti-Aβ antibodies for 1, 2 or 3 months. The second is an active vaccination study in which we examined 16 month old APPSw/NOS2-/- mice treated with Aβ vaccination for 4 months.

**Results:**

There is a significant activation of the MMP2 and MMP9 proteinase degradation systems by anti-Aβ immunotherapy, regardless of whether this is delivered through active vaccination or passive immunization. We have characterized this activation by gene expression, protein expression and zymography assessment of MMP activity.

**Conclusions:**

Since the MMP2 and MMP9 systems are heavily implicated in the pathophysiology of intracerbral hemorrhage, these data may provide a potential mechanism of microhemorrhage due to immunotherapy. Increased activity of the MMP system, therefore, is likely to be a major factor in increased microhemorrhage occurrence.

## Background

Alzheimer's disease (AD) is a progressive neurodegenerative disorder characterized clinically by a devastating cognitive decline. Pathologically, the three hallmarks of AD are extracellular amyloid plaques composed of aggregated Aβ peptide, intracellular neurofibrillary tangles composed of aggregated, hyperphosphorylated tau protein, and neurodegeneration characterized by loss of synapses and neurons [1]. The prevailing hypothesis for the pathological progression of AD is the amyloid hypothesis, and while it has undergone significant modification over the years, the basic thought is that the deposition of amyloid leads to neuronal changes causing the hyperphosphorylation of tau, which in turn leads to neuronal dysfunction and ultimately neuron death [2,3].

The amyloid hypothesis has been used as the basis of disease modifying therapies to treat AD. The most promising current pharmacological approach in clinical trials is anti-Aβ immunotherapy [4]. There are many ongoing clinical trials currently testing various modes of immunotherapy including active vaccination, passive immunization and IVIg therapy. While most data is very encouraging, including some promising cognitive outcomes [5] and evidence of amyloid reductions through PET imaging [6], there are vascular adverse events that remain a significant concern.

Vascular adverse events have been reported in both mouse studies [7] and in human clinical trials [5,8]. Immunotherapy has been shown to produce microhemorrhages (focused cerebral hemorrhagic events) and vasogenic edema (a pathological increase in extracellular fluid volume in the brain caused by damage to the blood brain barrier). Microhemorrhages have been shown in as many as twenty mouse studies to date and appear to require the presence of vascular amyloid deposition, a primary factor in cerebral amyloid angiopathy (CAA). The microhemorrhages occur regardless of whether the immunotherapy is active [9] or passive [10-13], yet the mechanism by which they occur is unknown. Vasogenic edema is an MRI phenomenon that has been observed in the ongoing bapineuzumab human clinical trial [6,14-16]. Its onset is more prevalent in ApoE4 carriers and its mechanism is unknown.

**Figure 1:**
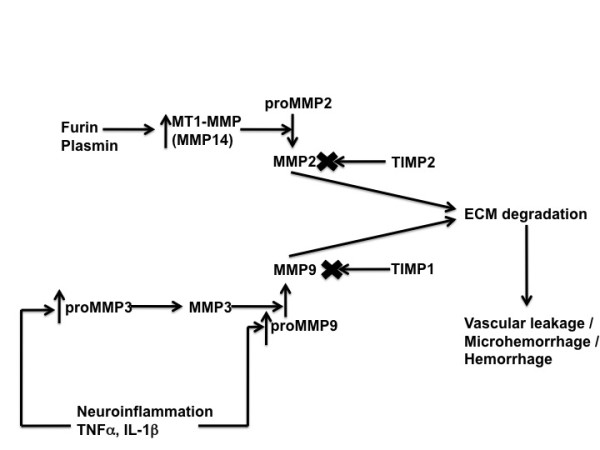
**Schematic of the MMP2 and MMP9 systems**. MMP2 is released in its pro form and cleavage by MT1-MMP leads to MMP2 activation. MT1-MMP is induced by furin and plasmin. TIMP2, tissue inhibitor of metalloproteinases 2, is an endogenous inhibitor of active MMP2. MMP3 is released in its pro form and cleavage leads to its activation. In turn, MMP9 is released in its pro form and MMP3 cleaves pro-MMP9 to produce active MMP9. Both pro-MMP3 and pro-MMP9 are induced by pro-inflammatory cytokines such as TNFα and IL-1β. TIMP1 is the endogenous inhibitor of active MMP9. Both MMP2 and MMP9 are known to degrade extracellular matrix proteins as well as tight junction proteins leading to vascular leakage and hemorrhage.

The focus of the current study was to examine changes in the MMP system in response to anti-Aβ immunotherapy. MMP2 and MMP9 are heavily implicated in the occurrence of cerebral hemorrhage [17-21], and MMP9 is induced by multiple inflammatory cytokines [22-24]. The MMP2 and 9 systems are complex, and involve a series of enzymatic cleavages to create the final active form of the enzyme (Figure 1). Briefly, MMP2 and MMP9 are released as a pro-enzyme that require enzymatic cleavage to be active. Pro-MMP2 is cleaved by MT1-MMP while pro-MMP9 is cleaved by MMP3. Both MMP2 and MMP9 have endogenous inhibitors, called TIMP1 and TIMP2 respectively (where TIMP = tissue inhibitor of metalloproteinases). We show that the MMP2 and MMP9 systems are activated following both passive and active anti-Aβ immunotherapy. The MMP changes only occur in mice that show significant microhemorrhage due to immunotherapy, indicating a critical role for the MMPs in the induction of microhemorrhage.

## Methods

### Transgenic mice

The study was approved by the Duke University Institutional Animal Care and Use Committee and conformed to the National Institutes of Health Guide for the Care and Use of Animals in Research. The APPSw mice (Tg2576, Swedish K760N/M671L [25]) were bred and housed by the Morgan-Gordon laboratory at the University of South Florida. The APPSw/NOS2^-/- ^mice were produced by crossing APPSw (Tg2576, Swedish K760N/M671L) transgenic mice [25] with NOS2^-/- ^(B6 129P2NOS2^tau1Lau^/J) mice (Jackson Laboratory, Bar Harbor, ME), as described previously [26].

### Active vaccination study

This study was previously published [9]. To summarize, APPSw/NOS2-/- and NOS2-/- mice aged 12 months were assigned to one of two treatment groups. Final sample sizes were 6 APPSw/NOS2^-/- ^mice receiving control vaccination, 6 APPSw/NOS2^-/- ^mice receiving Aβ vaccination, 6 NOS2^-/- ^mice receiving control vaccination and 6 NOS2^-/- ^mice receiving Aβ vaccination. All mice received 4 vaccinations over a 4 month period. Aβ and KLH vaccinations used complete Freund's adjuvant for the first innoculation and incomplete Freund's adjuvant for the remaining innoculations.

### Passive immunization study

This study was previously published [27]. To summarize, 19 month old APPSw mice were assigned to one of four groups, control antibody for 3 months or anti-Aβ antibody 2286 (Rinat Neurosciences, San Francisco CA) for one, two or three months (N = 4/group). The start of dosing was staggered such that all mice were the same age (22 months) at sacrifice. In the current study we examined four mice for each treatment group.

### Tissue processing

*Active vaccination study:* After injection with a lethal dose of ketamine, the mice were perfused intracardially with 25 ml normal saline. Brains were rapidly removed and bisected in the mid-sagittal plane. One half of each brain was immersion fixed in 4% paraformaldehyde, while the other was snap-frozen in liquid nitrogen and stored at -80°C. Frozen sections of the fixed hemibrain were collected following cryoprotection through sucrose. 25 μm sections were collected and stored in DPBS+sodium azide at 4°C until needed. The frozen hemibrain was pulverized using a mortar and pestle on dry ice. Brain powder was then stored at -80°C until needed. *Passive immunization study: *After injection with a lethal dose of pentobarbital, the mice were perfused intracardially with 25 ml normal saline. Brains were rapidly removed and bisected in the mid-sagittal plane. One half of each brain was immersion fixed in 4% paraformaldehyde, while the other was dissected into frontal cortex, posterior cortex, hippocampus, cerebellum and rest of remaining brain tissue. These pieces were flash frozen and stored at -80°C.

Eight 25 μm sections equally spaced 600 mm apart were selected from our active vaccination study for free floating immunohistochemistry for MMP9 (1: 1000, Rabbit polyclonal, Millipore, Billireca, MA) as described previously [27].

### Quantitative real-time RT-PCR

Approximately 40 mg frozen brain powder (for the active vaccination study) or the whole right hippocampus (for the passive immunization study) was used for RNA extraction using the PerfectPure RNA tissue kit (5 Prime Inc., Gaithersburg, MD). RNA concentrations were determined by UV spectrophotometry and cDNA produced using the cDNA archive kit (Applied Biosystems, Foster City, CA). Real-time PCR was performed using the TaqMan Gene Expression assay kit (Applied Biosystems, Foster City, CA) according to the manufacturer's instructions and as previously described [28]. All genes are normalized to 18 S rRNA. Normal non-transgenic mice served as the comparator and fold changes were calculated using the ^-delta delta Ct) ^method [29] The following genes were analyzed: 18 s (Hs99999901_s1), MMP2 (Mm00439498_m1), MMP3 (Mm00440295_m1), MMP9 (Mm00600163_m1), MT1-MMP (Mm00485054_m1), TIMP1 (Mm00441818_m1), TIMP2 (Mm00441825_m1).

### ELISA measurement

Active vaccination study only: Protein was extracted from 4 brains for each genotype using 100 mg pulverized brain powder in PBS with complete protease inhibitor (Sigma-Aldrich, St Louis MO) and quantified using the BCA protein assay kit (Pierce Biotechnology Inc. Rockford, IL, performed according to manufacturer's instructions). We used commercially available kits to assess MMP3, MMP9, TIMP1, MMP2 and TIMP2 and ran the assays according to manufacturer's recommendations (R&D Systems, Minneapolis, MN). All data were normalized to the total protein to yield ng/mg protein.

### Zymography

Enzymatic activities of tissue MMPs were measured using zymography in brain samples from the active vaccination study only: Protein was extracted using 100 mg pulverized brain powder in PBS and quantified immediately using the BCA protein assay kit (Pierce Biotechnology Inc. Rockford, IL. Performed according to manufacturer's instructions). Protein samples were immediately separated on a precast 10% gelatin zymogram gel (Invitrogen, Carlsbad, CA). The gel was removed, incubated in zymogram renaturing buffer for 30 minutes, equilibrated for 30 minutes in zymogram developing buffer at room temperature and then incubated overnight at 37°C with gently agitation in fresh zymogram developing buffer (all buffers obtained from Invitrogen, Carlsbad, CA). The next day, the gel was washed gently with water and incubated in Commassie blue (0.1% Commassie blue, 20% methanol, 10% acetic acid). The gel was stained for one hour and then destained using a 50% methanol, 10% acetic acid solution until clear bands were resolved (approximately 15 minutes). The gel was placed in a drying rack to dry and then imaged using the Kodak imager as described previously [30]. Two gels were necessary to include all samples. Densitometry was performed on the two gels and within each gel, data were normalized to the NOS2-/- band on the corresponding gel before being combined.

### BV2 microglial cell treatment

BV2 microglia were cultured as previously described [28]. The cells were split and plated into 6-well dishes and grown to confluency. The day prior to treatment we prepared the Aβ1-42 by bringing into solution according to manufacturers' directions (rPeptide, Bogart, GA). The Aβ was brought to a concentration of 5 μg/ml in PBS. This solution was then incubated at 37°C overnight to allow fibrils to form. To form an immune complex, Aβ aggregates in solution (5 μg/ml) were incubated with 10 μg/ml anti-Aβ monoclonal antibody 6E10 for 1 hr at 37°C, centrifuged at 100,000 × *g *for 30 min, and resuspended to original volume as described previously [31] Tau immune complexes were formed using the same methods, tau protein at a concentration of 5 μg/ml (rPeptide, Bogart, GA) was incubated with 10 μg/ml anti-tau monoclonal antibody (Clone T14, Invitrogen, Carlsbad, CA) for 1 hr at 37°C, centrifuged at 100,000 × *g *for 30 min and resuspended to original volume. Cells were then treated with 1 ml of the culture media containing 100 μl of either 2.5 μg/ml Aβ1-42, 5 mg/ml anti-Aβ IgG 6E10, the Abeta immune complex or the tau immune complex solution. Sister wells of BV2 cells were treated with fresh media changes at the same times as the treated cells and served as untreated controls. The cells were then incubated overnight. The following day the media was removed and the cells were washed with PBS and stored at -80°C until needed. qPCR and ELISAs were run in the same way as described above for the mouse samples.

### Analysis

Data are presented as mean ± standard error of mean (SEM). Statistical analysis was performed using the JMP statistical analysis program (SAS, Cary NC). Where appropriate, student's t test was performed. Statistical significance was assigned where the P-value was lower than 0.05.

## Results

Prussian blue is a histochemical staining method used to detect hemosiderin deposits, indicative of a location of a prior bleed. These data for both active vaccination and passive immunization have been previously published and are summarized in Table 1. A 10-fold increase was observed in the number of microhemorrhages in APPSw/NOS2-/- mice receiving Aβ vaccination as compared to APPSw/NOS2-/- mice receiving control vaccination (Table 1). Our previously published passive immunization study in APPSw mice also showed a significant increase in microhemorrhage occurrence [11]. The microhemorrhages following passive immunization occurred in a time dependent manner, where no increase was observed following one month of treatment, yet after two and three months of immunization a 6-fold increase in microhemorrhage was observed (Table 1). An increase in CAA was only observed in the passively immunized APPSw mice and we saw no change in CAA in the actively vaccinated APPSw/NOS2-/- mice. We believe this could be due to either the difference in mouse strain or the difference in age; the APPSw mice were significantly older and had significantly more CAA at the beginning of the study than did the APPSw/NOS2-/-.

**Table 1:** Summary of previously published data for anti-Aβ immunotherapy studies.

Study	Mouse model	Age (mo)	Histopathology			Cognition
				
			Aβ	CAA	Micro-hemorrhage	
Passive immunization	APPSw	19-22	↓60%	↑ 4-fold	↑ 6-fold	Improved

Active vaccination	APPSw/ NOS2-/-	12-16	↓85%	No change	↑ 10-fold	Reversed

**Figure 2:**
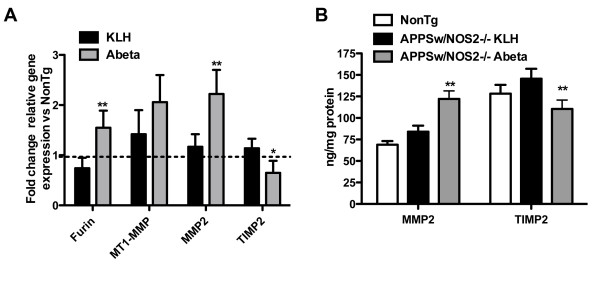
**Active Aβ vaccination in APPSw/NOS2-/- mice activates the MMP2 system**. Panel A shows the average #177; SEM) fold change in mRNA for components of the MMP2 system in APPSw/NOS2-/- mice vaccinated with either KLH or Aβ for 4 months. Furin and MMP2 are all significantly increased following vaccination while TIMP2 is significantly decreased. The dashed line indicates the average mRNA of the non-transgenic control mice. * indicates P < 0.05, ** indicates P < 0.01 compared to KLH vaccinated APPSw/NOS2-/- mice. Panel B shows the average #177; SEM) protein levels of MMP2 and TIMP2 as measured by sandwich ELISA. Data are normalized to the protein concentration of the tissue homogenate. ** indicates P < 0.01 compared to KLH vaccinated APPSw/NOS2-/- mice.

Quantitative real-time RT-PCR analysis of the levels of mRNA for the MMP2 system (Figure 2) in APPSw/NOS2-/- mice receiving KLH control vaccination showed no significant change compared to non-transgenic mice. In contrast, Aβ vaccination of APPSw/NOS2-/- mice significantly increased furin mRNA (a precursor to the activation of MMP2) but had no effect on MT1-MMP expression. Importantly, MMP2 mRNA levels were significantly increased compared to KLH treated mice while the endogenous inhibitor of MMP2, TIMP2, showed a significant decrease following Aβ vaccination (Figure 2A). To confirm that the gene expression data resulted in meaningful protein changes we performed ELISA measurements from brain lysate for MMP2 and TIMP2 using commercially available kits. The ELISA measurements confirmed a statistically significant increase in MMP2 following Aβ vaccination, while TIMP2 was significantly decreased in comparison to KLH control vaccinated APPSw/NOS2-/- mice (Figure 2B).

**Figure 3:**
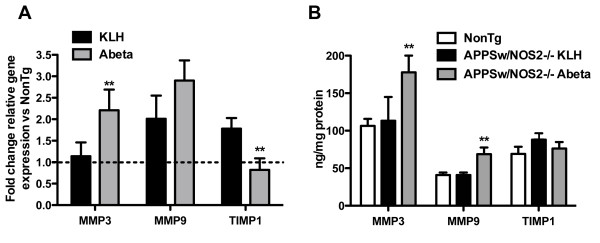
**Active Aβ vaccination in APPSw/NOS2-/- mice activates the MMP9 system**. Panel A shows the average (± SEM) fold change for components of the MMP9 system in mice vaccinated with either KLH or Aβ for 4 months. MMP3 and MMP9 are significantly increased. The dashed line indicates the average gene expression of the non-transgenic control mice. ** indicates P < 0.01 compared to KLH vaccinated APPSw/NOS2-/- mice. Panel B shows the average (± SEM) protein levels of MMP3, MMP9 and TIMP1 as measured by sandwich ELISA. Data are normalized to the protein concentration of the tissue homogenate. ** indicates P < 0.01 compared to KLH vaccinated APPSw/NOS2-/- mice.

The MMP9 system is also an important mediator of proteolytic activity that may lead to cerebral hemorrhage. Quantitative real-time RT-PCR analysis of the MMP9 system (see Figure 1) in APPSw/NOS2-/- mice receiving control vaccination showed that MMP9 and TIMP1 mRNA levels were increased compared to non-transgenic (NonTg) (Figure 3A). When compared to KLH control mice, MMP3 mRNA levels in Aβ vaccinated APPSw/NOS2-/- mice increased while MMP9 mRNA remained unchanged. In contrast, TIMP1 mRNA levels significantly decreased. Brain lysates were also analyzed for MMP3, MMP9 and TIMP1 protein levels as described in the methods. Both MMP3 and MMP9 levels were significantly increased in in APPSw/NOS2-/- mice following Aβ vaccination, while TIMP1 remained unchanged (Figure 3B). The sensitivities of the methods for measuring protein and gene expression are different and therefore it is not necessarily surprising that the MMP9 elevation in protein expression was not significant while the gene expression was and vice-versa for the TIMP1. It would be speculation at this point to suggest there may be significant differences between mRNA and translation to protein, however, this is a possibility. Importantly, is that the RNA and protein patterns remain consistent.

**Figure 4:**
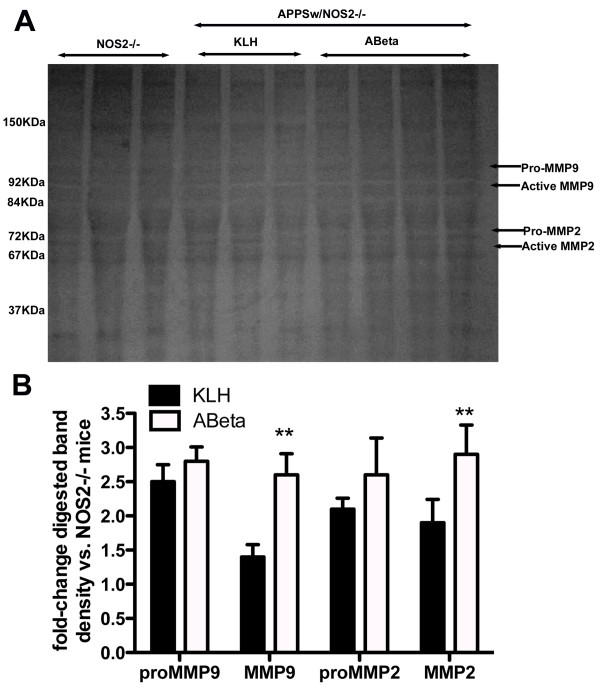
**Active Aβ vaccination in APPSw/NOS2-/- mice leads to increased activity of MMP2 and MMP9 as measured by gelatin zymography**. Panel A shows a representative image of the gelatin zymogram. The white bands indicate where the gelatin has been degraded by the proteinase. Based on the molecular weights of pro-MMP9, MMP9, pro-MMP2 and MMP2 we performed densitometry measurements on the white bands corresponding to these proteinases. Panel B shows the average (± SEM) densitometry quantification of the zymography bands. Data are shown as the fold-change from the average of the NOS2-/-densitometry data. Total sample size is N = 6 for NOS2-/-, N = 7 for KLH vaccinated and N = 7 for ABeta vaccinated. ** indicates P < 0.01 compared to KLH vaccinated.

While quantitative real-time RT-PCR and ELISA can provide estimates of the total RNA and protein expression levels of various MMPs, these methods do not provide a direct measure of the activity of these systems. Consequently, we performed gelatin zymography to assess the individual activity of MMP2 or MMP9. Band densities corresponding to the molecular weights of the pro and active forms of each enzyme were measured to provide semi-quantitative measurements of activity. As can be seen in Figure 4, bands indicative of gelatin degradation are observed at molecular weights of 67, 72, 84 and 92 KDa (Figure 4A). These correspond to active MMP2, pro-MMP2, active MMP9 and pro-MMP respectively. It is clear from examination of the gel that Aβ vaccinated APPSw/NOS2-/- mice show bands of a greater density corresponding to active MMP2 and active MMP9. Indeed, densitometry analysis showed a statistically significant increase in MMP2 and MMP activity in Aβ vaccinated mice compared to both NOS2-/- and control vaccinated APPSw/NOS2-/- mice (Figure 4B).

**Figure 5:**
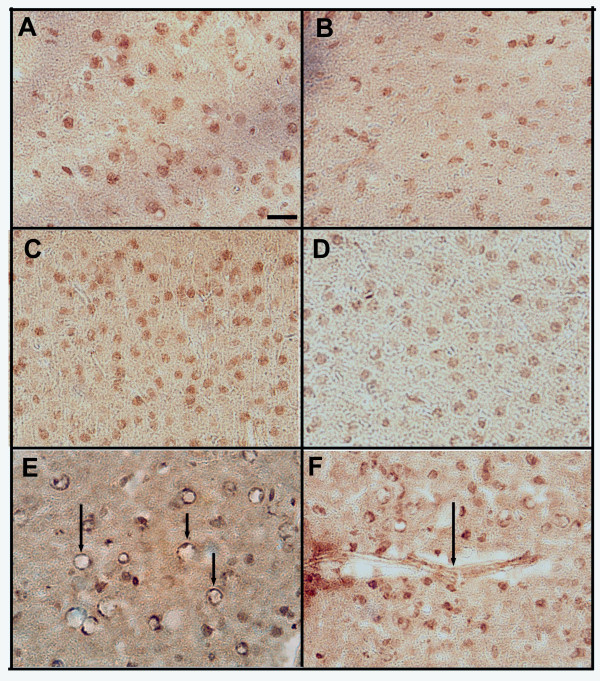
**Active Aβ vaccination in APPSw/NOS2-/- mice leads to increased total MMP9 expression at the cerebrovasculature**. Panels A and B show representative total MMP9 immunohistochemcial staining from non-transgenic mice. Lightly stained neurons can be found throughout the brain but there is no discernable vascular staining. Panels C and D show representative total MMP9 immunohistochemcial staining from APPSw/NOS2-/- receiving KLH vaccination for 4 months. Again, there are lightly stained neurons throughout the brain but no discernable vascular staining. Panels E and F show representative total MMP9 immunohistochemcial staining from APPSw/NOS2-/- mice receiving Aβ vaccination. In addition to the lightly stained neurons these mice show extensive MMP9 immunostaining at the cerebrovasculature, specifically capillaries (indicated by arrows in panel E) and penetrating arteries (indicated by the arrow in panel F). Panels A-F magnification = 200×, scale bar panel A for A-F = 25 μm.

To determine whether the cell-specific induction of MMP9 expression changed with Aβ vaccination, we examined the type of cell and the cellular pattern for total MMP9 expression using immunohistochemistry. Low intensity staining of neurons was observed in non-transgenic mice at 52 weeks of age. There was no detectable immunopositive staining in cells around the cereobrovasculature (Figure 5 panels A and B). APPSw/NOS2-/- mice receiving control vaccination showed largely the same staining pattern as NonTg mice, however, the staining intensity of neurons appeared slightly greater (Figure 5 panels C and D). Following Aβ vaccination robust expression of MMP9 was found in association with the vasculature in all Aβ vaccinated APPSw/NOS2-/- mice. Figure 5E shows extensive staining of capillaries in the parietal cortex, highlighted by arrows. In addition, Figure 5F shows an example of an intensely stained larger penetrating arteriole in the frontal cortex of an Aβ vaccinated APPSw/NOS2-/- mouse.

**Figure 6:**
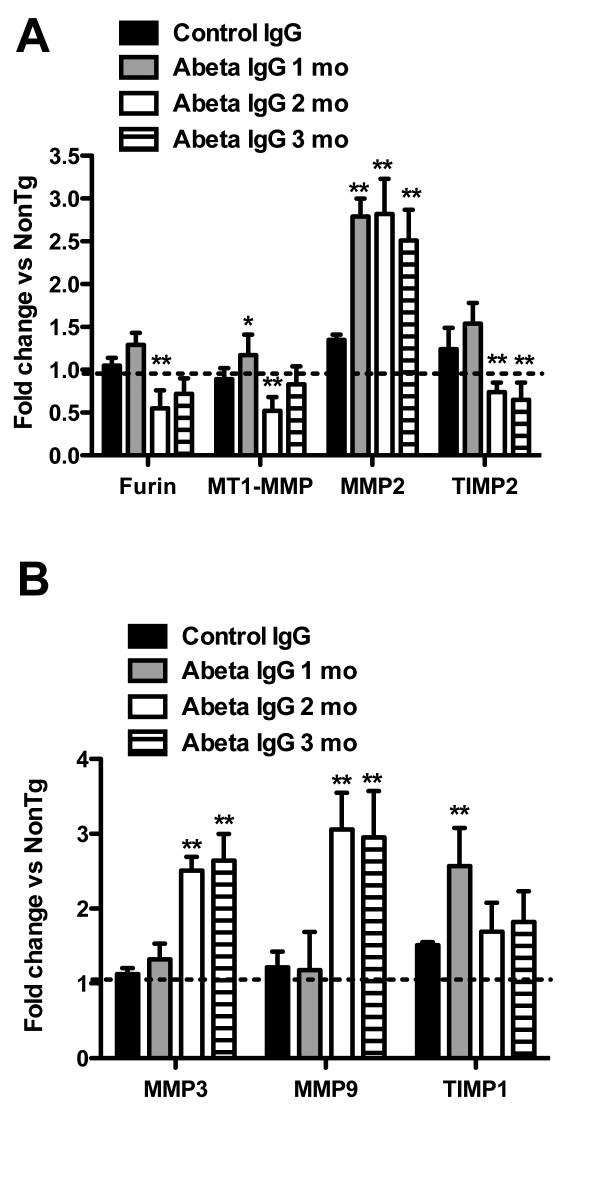
**Passive immunization in APPSw mice leads to a rapid activation of the MMP2 system as well as a later activation of the MMP9 system**. Panels A and B show relative gene expression analysis of the hippocampus of APPSw mice receiving control antibody for 3 months or anti-Aβ antibody for 1, 2 and 3 months. Data are shown as the average (± SEM) fold change in mRNA. Panel A shows data for the MMP2 system, panel B shows data from the MMP9 system. * indicates P < 0.05, ** indicates P < 0.01 compared to control antibody treated mice.

In order to confirm that the changes in the MMP systems were due to anti-Aβ immunotherapy and not unique to our study in APPSw/NOS2-/- mice, we performed quantitative real-time RT-PCR of APPSw mice that had been passively immunized and shown to have significant Aβ reductions, cognitive improvements and inflammatory changes (Table 1 and [27]). We examined components of both the MMP2 and MMP9 systems and found strikingly similar patterns to the data in the actively vaccinated APPSw/NOS2-/- mice. Interestingly, the MMP2 system showed changes that were dependent on the duration of passive immunization treatment. Following one month of immunization, APPSw mice showed increased gene expression of MMP2 that remained elevated at 2 and 3 months (Figure 6A). After a slight elevation at 1 month, furin levels transiently decreased thereafter while TIMP2 mRNA levels significantly decreased from the 1 month time point. Thus after two months of immunization which is the time at which we observe significant microhemorrhage under these conditions, we find that MMP2 gene expression remains significantly elevated, while furin and TIMP2 are significantly decreased (Figure 6A). If protein expression and activity follows the changed gene expression, these results would suggest an increased enzymatic activity of MMP2 due to less inhibitor. Finally, the MMP9 system is also activated following passive immunization. Interestingly, following one month of immunization, gene expression of MMP3 and MMP9 are not largely affected, yet there is a significant increase in the MMP9 inhibitor, TIMP1 (Figure 6B). Importantly, we do not observe microhemorrhage at this time point. By two months of immunization MMP3 and MMP9 are significantly increased and although not reaching statistical significance, TIMP1 is reduced compared to the one month time point (Figure 6B). This pattern is also retained for the three months of immunization.

**Figure 7:**
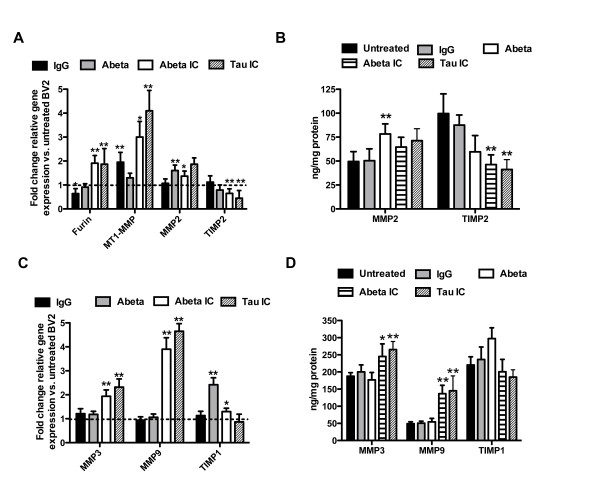
**Treatment of BV2 microglial cells with immune complexes leads to induction of components of the MMP2 and MMP9 systems**. Panels A and C show the average (± SEM) mRNA levels of the MMP2 (A) and MMP9 (C) systems in BV2 cells treated with IgG, Abeta, Abeta immune complexes (Abeta IC) or tau immune complexes (tau IC) for 24 hours. Data are shown as fold change compared to untreated sister BV2 cultures. * indicates P < 0.05, ** indicates P < 0.01 compared to untreated BV2 cells. Panels B and D show protein expression as measured by sandwich ELISA for components of the MMP2 (B) and MMP9 (D) systems. * indicates P < 0.05, ** indicates P < 0.01 compared to untreated BV2 cells.

Finally, to establish if MMP expression was induced via Fcγ-receptor signaling we treated BV2 microglial cultures with anti-Aβ IgG alone, Aβ1-42 fibrils, anti-Aβ IgG/Aβ immune complexes or anti-tau/tau immune complexes and compared the gene expression changes for various MMPs. Three separate culture groups were tested and separate wells of the same culture were used to extract RNA for gene expression analysis and protein for ELISA measurements. We found that IgG treatment of BV2 cells selectively stimulated the induction of MT1-MMP of the MMP2 system as determined by qRT-PCR (Figure 7A). No component of the MMP9 system was activated by IgG treatment (Figure 7C and 7D). Aβ fibril treatment stimulated both MT1-MMP and MMP2 of the MMP2 system as measured by qRT-PCR (Figure 7A), ELISA measurement of MMP2 confirmed this change (Figure 7B). Aβ treatment increased TIMP1 expression significantly but no other changes were noted with respect to the MMP9 system (Figure 7C and 7D). Interestingly, treatment of BV2 cells with anti-Aβ IgG-Aβ or anti-tau IgG-tau immune complexes resulted in a significant reduction of TIMP2, the inhibitor of MMP2 at the RNA and protein level, and also activation of the MMP9 system including increased expression of MMP3 and MMP9 as measured by qRT-PCR and ELISA (Figures 7C and 7D). The treatment with immune complex, regardless of whether this was Aβ immune complex or tau immune complex, was the only treatment to activate the MMP9 system indicating a requirement for Fcγ-receptor signaling through opsonized antigen.

## Discussion

Microhemorrhages are a significant consequence of cerebral amyloid angiopathy (CAA) and based on MRI detection, are known to occur in as many as 40% of AD cases [32]. As imaging techniques become more sensitive, this percentage may become even higher. In addition, microhemorrhages are exacerbated by anti-Aβ immunotherapy in mouse and human studies [8,10-13]. Another vascular adverse event of immunotherapy is vasogenic edema, which has been found in the ongoing immunotherapy trial of bapineuzumab [5]. Vasogenic edema involves disruption of the blood-brain barrier and may therefore share a common mechanism of action with microhemorrhages. While a number of pathophysiological factors may contribute to microhemorrhage and vasogenic edema, proteolytic destabilization of the neurovascular unit may be an important feature. Matrix metalloproteinases are a family of proteinases known to degrade components of extracellular matrix, as well as other proteins including cytokines and pro/anti-angiogenic modulators. In particular, MMP2 and MMP9 are implicated in cerebrovascular dysfunction. Using mouse models of AD, we have examined changes in these MMP systems with immunotherapy in two separate studies, an active vaccination study and a passive immunization study, both of which have shown increased incidence of microhemorrhage.

MMP2 is also known as gelatinase A and is secreted as a pro-form, which requires cleavage by MT1-MMP (MMP14) for its activation [33]. MT1-MMP is activated by furin and plasmin. In addition, plasmin has been shown to directly activate surface-bound pro-MMP2 [34]. An endogenous inhibitor of MMP2, TIMP2, controls the activity of MMP2 post-translationally [35]. Importantly, MMP2 has been shown to be involved in the early opening of the blood brain barrier following cerebral reperfusion [36,37]. This MMP2-mediated process involves degradation of the tight junction protein, claudin-5 [37].

In order to evaluate the activity of the MMP2 system in our mouse models of AD, we examined the gene and corresponding protein expression levels of furin, MT1-MMP, MMP2 and TIMP2 in mice that received active Aβ vaccination. We found that vaccination resulted in increased gene expression of furin, and MMP2 along with a concomitant decrease in TIMP2 expression, while protein expression followed the same pattern. In addition, we established that MMP2 activity was significantly increased by performing gelatin zymography on brain lysates. These observed changes are consistent with an increase in proteolytic activity that may degrade collagen or other extracellular matrix proteins that comprise the blood brain barrier, leading to leakage of cerebral vessels. To confirm that our findings were relevant to immunotherapy, we obtained frozen hippocampi from APPSw mice that were passively immunized and showed significant microhemorrhage incidence [11,27]. Indeed we found similar trends in passively immunized mice as compared to the actively vaccinated mice. Following one month of immunization MMP2 was increased and for the following two and three months only the MMP2 remained significantly elevated. TIMP2 levels, however, transiently and slightly increased at 1 month then remained at a reduced level. While it may seem that the active vaccination had a lesser effect on the MMP2 system than the passive immunization, it is important that we not draw conclusions from this since these are different strains of mice (APPSw/NOS2-/- vs. APPSw) and different aged mice (16 vs. 22 months). All of these things could account for the apparent smaller effect size in the active vaccination study. Overall, the MMP2 system data suggest that increased activity of the MMP2 system may be involved in abnormal proteolytic activity at the neurovascular interface.

The second major proteolytic enzyme in the brain, MMP9 (also known as gelatinase B), has been heavily implicated in many types of CNS injury including stroke [38], ischemia [35] and trauma [39]. The normal function of MMP9 is degradation of extracellular matrix to allow for cell migration and also degradation of basement membranes to allow for cell movement across the blood vessels. In addition, Aβ is a natural substrate of MMP9 indicating a role in Aβ homeostasis [40]. MMP9 is secreted in a pro form, which is cleaved to the active MMP9 by other MMPs, primarily MMP3. Inflammatory cytokines such as IL-8 and TNFα [22,23] are known to be involved in the regulation of the MMP9 system through the induction of both MMP3 and MMP9. Also, TNFα [41] and IL-1β [42] are examples of cytokines that are actually activated by MMP9. Similar to MMP2, an endogenous inhibitor of the proteolytic activity of MMP9 has been described, and is known as TIMP1. TIMP1 provides an additional level of regulation of proteolysis in the brain and can be regulated, in turn, by inflammatory factors and tissue redox balance [35,43].

The MMP9 system was assessed by qRT-PCR, ELISA, zymography and immunohistochemistry in our Aβ vaccination study in APPSw/NOS2-/- mice. We found that MMP3 gene and protein expression are robustly increased, while MMP9 is increased to a lesser extent. TIMP1 levels were reduced at the gene expression level and remain unchanged at the protein level. A similar pattern was observed at 2 and 3 months in the passive immunization study. In addition, zymography revealed that MMP9 activity is significantly increased following Aβ vaccination in APPSw/NOS2-/- mice. Finally, MMP9 immunohistochemistry showed a dramatic elevation of expression in the endothelial cells of the cerebrovasculature in APPSw/NOS2-/- mice receiving Aβ vaccination compared to those mice control vaccination.

In vitro MMP2 and MMP9 have both been shown to proteolyze Aβ [44,45]. Moreover, in vivo MMP9 is increased in association with amyloid plaques in transgenic mouse models of amyloid deposition [46]. Additionally, treatment of APP/PS1 mice with FK506 lowered Aβ and increased MMP9 suggesting a relationship between elevated MMP9 and lower Aβ [47]. However, in human AD despite MMP9 being overexpressed around amyloid deposits [48], MMP2, MMP3 or MMP9 cannot be associated with plaque load in AD suggesting that they are not crucial for the regulation of plaque load [49]. We believe that the upregulation of MMPs in our immunotherapy studies may play a small role in the clearance of Aβ due to immunotherapy but other clearance mechanisms are also known to play a significant role (reviewed in [4]). Our data in BV2 cells indicates that MMPs, especially the MMP9 system can be upregulated through Fcγ receptor signaling mechanisms more efficiently than Aβ alone and this is likely the primary mechanism for MMP upregulation in our immunotherapy studies.

We have previously shown a robust inflammatory response to anti-Aβ immunotherapy, regardless of whether an active vaccination or passive immunization approach is used [50-54]. To determine whether the inflammatory response to immunotherapy was, at least in part, responsible for the increased MMP2 and MMP9 activities, we used BV2 microglial cells to determine how immune complexes change the MMP expression. Treatment of BV2 microglial cells with immune complexes of anti-Aβ IgG/Aβ or anti-tau/tau significantly increased MMP3 and MMP9, the two MMPs most closely associated with inflammation. In contrast, we did not observe significant changes in MMP3 or MMP9 with IgG alone or Aβ alone, indicating an Fcγ-receptor mediated activation.

## Conclusions

Overall, we believe that the inflammatory response to anti-Aβ immunotherapy leads to increased MMP activity by increasing expression of two key proteolytic systems in the brain. The elevation of MMP2 and MMP9 levels and activity coupled with the restricted levels of endogenous tissue inhibitors TIMP1 and TIMP2 strongly suggests a shift in the MMP/TIMP ratio toward favoring active proteolysis. Degradation of vascular basement membranes and tight junction proteins are likely results of such a shift. These data, plus published data describing the dependence of cerebral hemorrhage on MMP9 activity, indicate that increased MMP activity and resulting protein degradation may be a factor in the increased incidence of microhemorrhage found with anti-Aβ immunotherapy.

## List of abbreviations

Aβ: beta-amyloid; AD: Alzheimer's disease; APP: Amyloid precursor protein; CAA: Cerebral amyloid angiopathy; KLH: Keyhole limpet hemocyanin; MMP: matrix metalloproteinase; NOS2: Nitric oxide synthase 2; TIMP: Tissue inhibitor of metalloproteinases.

## Competing interests

The authors declare that they have no competing interests.

## Authors' contributions

DMW performed all experiments, data analysis and interpretation, and wrote the manuscript. DM and MNG contributed the passive immunization tissue and provided direction for the performance of the studies, as well as editing the manuscript. TLT performed the BV2 experiments with immune complexes and analyzed the data. LAR and DAW provided essential guidance for the performance of the studies on MMPs, in particular the zymogram studies. CAC provided guidance on the performance of studies, data interpretation and editing of the manuscript. All authors have read and approved the final version of the manuscript.
